# The source of cell-free mitochondrial DNA in trauma and potential therapeutic strategies

**DOI:** 10.1007/s00068-018-0954-3

**Published:** 2018-04-09

**Authors:** Kabilan Thurairajah, Gabrielle Daisy Briggs, Zsolt Janos Balogh

**Affiliations:** Department of Traumatology, John Hunter Hospital and University of Newcastle, Newcastle, NSW Australia

**Keywords:** MtDNA, Inflammation, Trauma, Therapeutics

## Abstract

Mitochondria play a key role in the pathophysiology of post-injury inflammation. Cell-free mitochondrial DNA (cf-mtDNA) is now understood to catalyse sterile inflammation after trauma. Observations in trauma cohorts have identified high cf-mtDNA in patients with systemic inflammatory response syndrome and multiple organ failure as well as following major surgery. The source of cf-mtDNA can be various cells affected by mechanical and hypoxic injury (passive mechanism) or induced by inflammatory mechanisms (active mechanism). Multiple forms of cf-mtDNA exist; mtDNA fragments, mtDNA in microparticles/vesicles and cell-free mitochondria. Trauma to cells that are rich in mitochondria are believed to release more cf-mtDNA. This review describes the current understanding of the mechanisms of cf-mtDNA release, its systemic effects and the potential therapeutic implications related to its modification. Although current understanding is insufficient to change trauma management, focussed research goals have been identified to pave the way for monitoring and manipulation of cf-mtDNA release and effects in trauma.

## Introduction

Up until recently, practical clinicians have regarded DNA and mitochondria as intracellular structures, unrelated to the pathophysiology of trauma. We are now beginning to understand the impact of cell-free mitochondrial DNA (cf-mtDNA) on post-traumatic inflammation. Cf-mtDNA are fragments of mitochondrial DNA within the extracellular space. In this review, we consider this to include exposed cf-mtDNA fragments, mitochondrial DNA (mtDNA) contained within vesicles and microparticles or extruded whole mitochondria. cf-mtDNA gained interest as a trigger of inflammation due to its similarity to bacterial DNA, its ability to stimulate innate immune responses through Toll-like receptor 9 (TLR-9) and its ability to induce tissue injury when injected in animal models [1, 2]. Numerous pathological states such as after trauma, cancer, mental illness and degenerative disease have been associated with measurable increases of plasma mtDNA [3–9]. The current hypothesis surrounding cf-mtDNA in trauma is that significantly severe trauma results in high concentrations of mtDNA release from tissues with subsequent uncontrolled inflammation and end-organ damage, with numerous studies reporting associations between cf-mtDNA and post-injury complications [4, 10, 11]. Given the myriad of pathophysiological processes occurring after traumatic injury, mtDNA could enter the circulation from a range of tissue types and via numerous mechanisms, some of which are due to the tissue injury itself, through post-injury inflammatory mechanisms or through interventions during their hospital stay. This review highlights both described and hypothetical sources of cf-mtDNA in the context of traumatic injury and subsequent patient management and highlights potential therapeutic strategies targeting mtDNA.

## Mitochondria and mtDNA

Polytrauma is associated with a severe systemic inflammatory response, which can lead to organ dysfunction and failure. Here, mitochondria have been in the centre of attention for long time. The focus on mitochondria in shock, sepsis and organ failure-related catabolic states was obviously related to their ATP production and supply of energy to the high-demand post-injury state and inflamed organs. Traumatic shock resuscitation strategies were aimed at maximising oxygen delivery to optimise the oxygen consumption of the hypercatabolic state after severe injury. The goal is to prevent tissue hypoxia.

Mitochondria are often over-simplified as cellular energy generators. While carrying out oxidative phosphorylation, mitochondria also facilitate decomposition of reactive oxygen species (ROS), calcium homeostasis, thermoregulation and cell death amongst other functions [12, 13]. Our mitochondrial genome is uniquely and exclusively of maternal origin [14].

It is imperative to recognise the bacterial origin of mitochondria to understand their immunogenic potential. The huge evolutionary step from prokaryotes to eukaryotes can be characterised by the energy production and the presence of mitochondria. The evolution and origin of eukaryotic cells is a debated topic but a very probable hypothesis is the fusion of the archaebacteria and ancient proteobacteria. In this symbiosis the archaebacterium became the energy centre of the proteobacterium and the new organism could be considered as the ancient protozoon. Having initially lived as a symbiont in eukaryotic cells, the mitochondrial genome gradually shrunk to the size of 16.5 kilobase pairs but retained its circular structure and abundant CpG motifs [12, 15]. The similarity to bacterial DNA is partly responsible for activating the immune system [16], namely through TLR-9. MtDNA itself causes sepsis-like symptoms in SIRS in a similar manner to bacterial molecules and these phenomena are difficult to clinically differentiate. In addition to its DNA, mitochondria contain multiple damage-associated molecular patterns (DAMPs) which include cardiolipin, transcription factor A (TFAM), *N*-formyl-peptides, adenosine triphosphate (ATP) and cytochrome c [15, 17, 18].

The mitochondrion consists of an outer phospholipid membrane containing nuclear-encoded proteins and a cardiolipin-rich and highly complex inner membrane where oxidative phosphorylation takes place. MtDNA is contained within the inner membrane of mitochondria and protected from contact with immune cells. MtDNA is tightly bound to and essentially coated in the aforementioned mtDAMP, TFAM [19] that packages mtDNA into nucleoid structures. The copy number of mitochondrial genomes per mitochondria varies with different tissue types and other factors such as age [20] and in diseases such as cancer [21] and has been reported between 2 and 10 copies per mitochondrion.

Not all cell types have equal mitochondrial content. Cells with high energy demands generally have higher mitochondrial content, although the direct evidence in this area, especially in human tissues is scarce. Tissue types known to have high mitochondrial content include skeletal muscle, smooth muscle, hepatocytes and renal tubular cells. Typically, skeletal muscle cells contain approximately 5000 mitochondria per cell while platelets contain 4 mitochondria per cell. Conversely, erythrocytes contain no mitochondria [22]. It should be noted that in some cells/cell cycle stages, mitochondria can also exist as a continuous filamentous structure with the ability to fragment into smaller mitochondria [23], so number is not necessarily a true indication of mitochondrial mass. This ideology suggests that trauma to skeletal muscle or liver releases more mtDNA than trauma to skin or bone.

Under physiologic conditions, cell turnover in the form of apoptosis does not result in release of free mtDNA into the extracellular space. Cell contents are normally packaged into apoptotic bodies that become phagocytosed without ever leaking directly into the extracellular space [12, 24] and, therefore, do not trigger immune responses. The process of mitophagy involves the breakdown of individual mitochondria due to nutrient deprivation (type I mitophagy) or mitochondrial damage (type II mitophagy). Here too, mtDNA and other mtDAMPs are not exposed to cytosolic pattern recognition receptors, but degraded safely within mitophagosomes. On the smaller scale, Type III mitophagy or micromitophagy involves mitochondria ridding themselves of damaged components (such as oxidised proteins) via packaging into vesicles, release into the cytosol and fusion with lysosomes [25]. Type III mitophagy is hypothesised to contribute to inflammation following trauma and is described later in this review (“Mitochondrial DNA: pathways to inflammation”).

## Natural history of cf-mtDNA release and its tissue origin

Most of our knowledge related to the presence of cf-mtDNA in clinical trauma scenarios is limited by the broad categorisation of “circulatory/plasma” concentrations. The true natural history of cf-mtDNA is hypothesised to vary with the nature of injury and speed of recovery. The hypothesis also sees increased cf-mtDNA associated with SIRS and normalisation of cf-mtDNA associated with subsequent clinical recovery. Associations between injury severity and concentration of plasma mtDNA have been observed in trauma cohorts; however, the data are not standardised and forming systematic conclusions is not possible. Plasma mtDNA has been detected as early as 20 min post-injury [26]. However, only two studies have performed serial measurements of plasma mtDNA. The observed patterns of cf-mtDNA release varied with McIlroy et al. showing early increase in concentration post-injury, transient reduction of concentration post-surgery, followed by gradual rise till Day 5 post-injury [27]. Yamanouchi et al., however, found an increase and rapid decrease in mtDNA concentration on day 1 post-injury with a lower plateau from days 2 till 5, however, still significantly elevated from control [28].

The anatomical source of cf-mtDNA or its specific cellular/tissue origin in different post-injury scenarios is largely unknown. The large quantities and ever-increasing plasma concentrations after trauma or surgical interventions makes us hypothesise that mitochondria-rich cells or cells in abundancy at the site of injury produce them. Cells that migrate to sites of injury include PMN, platelets and stem cells. These cells can all release cf-mtDNA and are involved in mtDNA-initiated inflammation. It is plausible that tissue injury results in an immediate surge of cf-mtDNA into the extracellular space which results in secondary active release of cf-mtDNA by immune cells and platelets. This secondary active release of cf-mtDNA is thought to be independent of cell necrosis. This hypothesis supports the observations of McIlroy et al. of continued increase in plasma mtDNA concentration, days after injury, with no associated increase in cell necrosis markers.

## Release of cf-mtDNA via cell necrosis in trauma and surgery

Cell necrosis represents the archetypal mode of mtDNA release that occurs with trauma. Tissue injury results in uncontrolled rupture of cell membranes and cell contents are spilled into the extracellular space [24, 29]. Along with mtDNA, numerous other DAMPs and ROS are released which damage neighbouring cells leading to more cell necrosis and mtDNA release [15, 18]. Theoretically, the concentration of mtDNA is highest at the site of injury where cf-mtDNA gradually drifts into capillaries and lymphatics to become circulating cf-mtDNA. Cells with higher mitochondrial content, when necrotised, naturally release more cf-mtDNA. The more cf-mtDNA released, the more potential for pro-inflammatory activation. As seen in observational studies on trauma patients, cf-mtDNA concentration positively correlated with injury severity, incidence of systemic inflammatory response (SIRS) and mortality [11, 26, 30].

Cf-mtDNA has been implicated in inflammation associated with ischaemia–reperfusion injury [2, 31] and furthermore, a study of myocardial ischaemia at a cellular and organ level demonstrated a positive correlation between cf-mtDNA concentration and injury severity [32]. Zhang et al. demonstrated raised plasma mtDNA concentration in rats that underwent haemorrhagic shock and trauma [17]. This mtDNA concentration was raised throughout the 7 days of sampling. No studies have demonstrated the effect of isolated shock on cf-mtDNA. Strenuous exercise has also been shown to induce cf-mtDNA release. Stawksi et al. found increased cf-mtDNA immediately post-exercise; however, a decrease in cf-mtDNA baseline occurred with repeated exercise. The mechanism of this release is unclear.

Reperfusion of injured tissue is a delayed means of cf-mtDNA reaching circulation after trauma. This hypothesis of extracellular accumulation of cf-mtDNA prior to its detection in circulation is also supported by the observation of Martinez-Quinones et al. in patients who underwent open abdominal surgery. Peritoneal lavage was associated with lower plasma mtDNA concentrations [33]. This demonstrates surgical reduction of extracellular mtDNA load prior to its entry into circulation and raises the possibility of timely mtDNA removal as a potential therapy.

Surgery can also increase cf-mtDNA release. Surgical intervention results in tissue injury with a resultant cf-mtDNA surge. McIlroy et al. observed higher concentrations of plasma cf-mtDNA in patients who underwent major pelvic surgery compared to minimally invasive surgery [27]. Hauser et al. studied the content of femoral reamings and identified high quantities of mitochondrial DAMPs [34]. Likewise, Sandler et al. found elevated concentrations of mtDNA in patients following cardiopulmonary bypass surgery. Interestingly, the higher concentrations of mtDNA correlated with post-operative complications [35].

Increased cf-mtDNA concentrations are also associated with turbulent blood flow through extracorporeal membrane oxygenation (ECMO) devices. Bynum et al. found increasing concentrations of cf-mtDNA with high flow rates and cycle durations of ECMO therapy. The exact mechanism of mtDNA release in this setting was not investigated. Turbulent flow may have resulted in activation of clotting cascades or lysis of cells [36, 37]. Similarly, endotracheal tube placement in surgery has been associated with higher concentrations of mtDNA in throat lavage fluid. Puyo et al. found a correlation between non-infected sore throat post-intubation and elevated throat lavage mtDNA concentration [38].

**Fig. 1:**
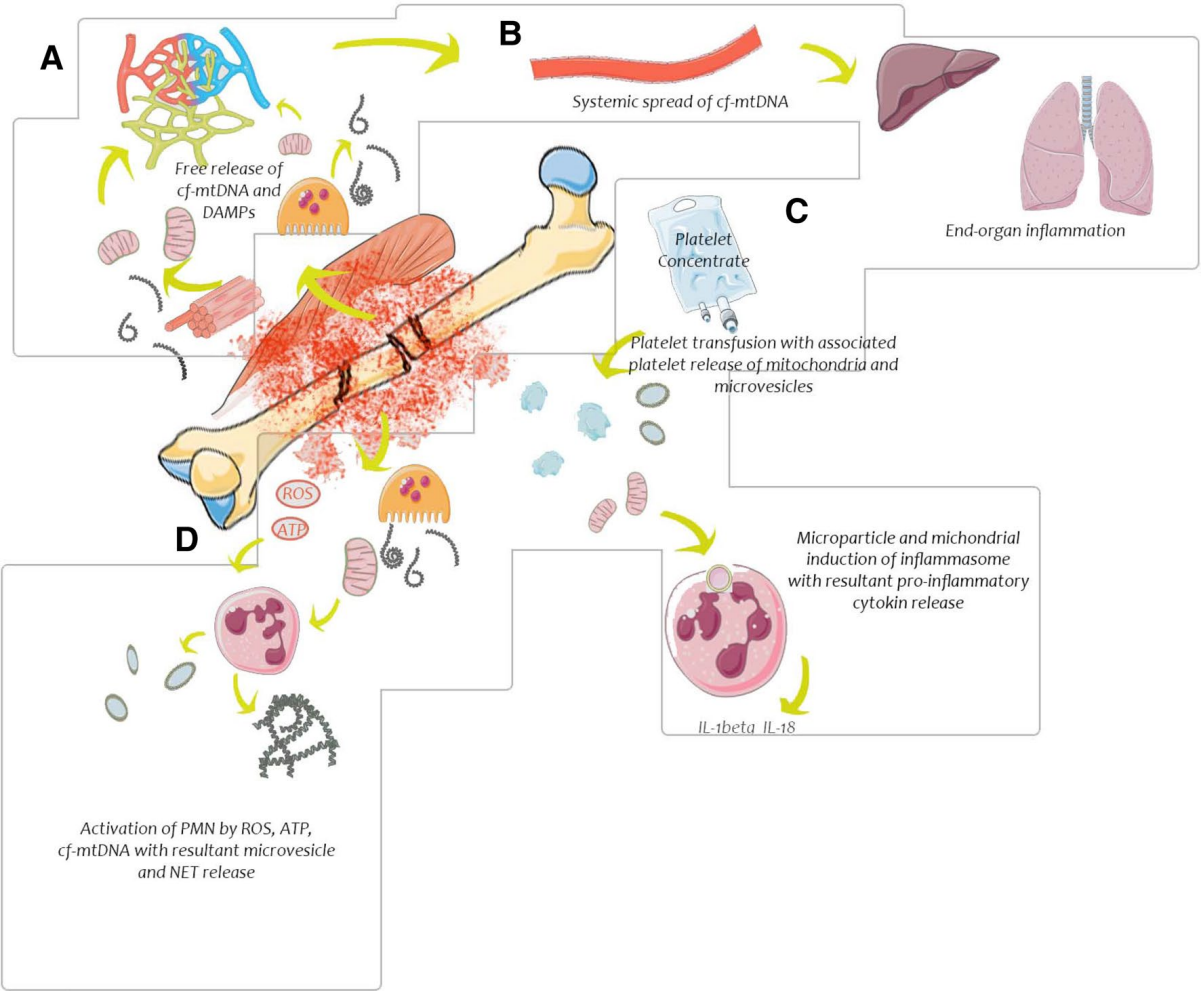
Cell-free mitochondrial DNA in trauma. This figure shows the cellular response to tissue injury. **a** In the immediate area of injury, cf-mtDNA is release from injured cells. **b** Transport of cf-mtDNA occurs through circulation to remote areas resulting in end-organ injury. **c** Platelet concentrate infusions contain activated platelets with cf-mtDNA and increase the cf-mtDNA load. **d** Generation of ATP, ROS in combination with release of mitochondrial DAMPs activate immune cells and platelets to release inflammatory cytokines

These forms of mtDNA release are thought to represent uncontrolled mtDNA release from within mitochondria to the extracellular space by rupture of cell membranes from mechanical trauma. While this is clearly a major source of mtDNA post-injury, there are alternative sources of post-injury cf-mtDNA that can arise from active release from cells, which involve a more complex mechanism of first cytosolic then extracellular release (Fig. 1).

## Active release of MtDNA

### Microparticles/vesicles

Active and passive mtDNA release are closely linked by ROS generation [12]. ROS are normally reduced by mitochondria but when excessive amounts accumulate, as occurs with severe tissue injury and passive cf-mtDNA release, mitochondria begin to swell and lyse. MtDNA do not have histones, unlike nuclear DNA, and in turn are susceptible to oxidative injury from ROS [39]. Oxidised mtDNA then accumulates in the cell cytosol [12, 40] and is packaged into microparticles to be extruded to favour cell survival [40]. Caielli et al. postulated that this phenomenon occurs when neutrophils are unable to degrade damaged mitochondria through mitophagy [40]. Cytosolic mtDNA accumulates and this has also been shown to trigger cell injury [41, 42]. This is likely a form of Type III mitophagy with formation of microparticles/vesicles.

Microparticles are small plasma membrane-encapsulated structures that are released from cells. They have been shown to contain mitochondrial elements including mtDNA [43]. The function of these released microparticles is not completely understood. Hepatocytes in patients with non-alcoholic steatohepatitis release oxidised mtDNA in microparticles which are engulfed by polymorphonuclear cells (PMNs). Intracellularly, they activate Toll-like receptor-9 (TLR9) and induce pro-inflammatory inflammasome production [44].

Platelets are able to release microparticles containing functional mitochondria [22, 45, 46]. Boudreau et al. found high levels of cf-mtDNA in patients who developed transfusion reactions from platelet concentrates. In vitro, platelets were shown to release mitochondria in microparticles and as free mitochondria when activated with thrombin or immunoglobulin. The mitochondria served as substrates for co-secreted phospholipase A2. Upon digestion of the encapsulating membranes by phospholipase A2, cf-mtDNA was released into the extracellular space and induced inflammation [22]. This represents a mechanism of cellular mitochondrial exocytosis without necrosis.

Marcoux et al. followed on to investigate concentrations of cf-mtDNA in stored platelets. Platelet-rich plasma (PRP) was found to contain higher amounts of cf-mtDNA than buffy coat and apheresis platelet concentrates. Again the cf-mtDNA was in the form of mitochondria within microparticles and free mitochondria [46]. These microparticles are pro-inflammatory as discovered by Mobarrez et al. who studied plasma from patients with active systemic lupus erythematosus. Inflammation correlated with observation of increased concentrations of circulating mitochondria within microparticles. Some microparticles were lined with immunoglobulins that activated inflammatory cascades, thus ultimately resulting in lysis of microparticles and release of cf-mtDNA [45]. These microparticles can also be engulfed by neutrophils to stimulate pro-inflammatory changes [22, 47].

While there is ample evidence that cf-mtDNA is increased following injury and has associations with poor outcomes, the form of the cf-mtDNA, i.e. free, microparticle or mitochondria encapsulated has not been described for trauma patients. The clinical relevance of these potential forms of cf-mtDNA cannot be understated; post-injury platelet activation and transfusion of stored platelets represent likely candidates for cf-mtDNA generation beyond injury itself and a greater understanding of post-injury cf-mtDNA profile may lead to new therapeutic targets or changes to blood product processing with the view to reduce immunogenicity.

## Active release: free mitochondrial exocytosis

As discussed above, platelets are able to excrete mitochondria as free organelles into the extracellular space [22, 46]. This feature is not exclusive to platelets. Maeda et al. demonstrated that cells release free mitochondria into the extracellular space when reacting to tumour necrosis alpha (TNF-alpha) stimulation. This is a part of cell necroptosis and the released mitochondria triggers pro-inflammatory changes in immune cells [48]. Given TNF-alpha is also implicated in reperfusion injury, it is plausible that this phenomenon occurs with reperfusion injury.

Mesenchymal stem cells (MSCs) also release mitochondria when under oxidative stress. This is a survival tactic that allows mitophagy of damaged mitochondria to be outsourced to nearby macrophages. The free mitochondria may, however, trigger inflammation in the recipient macrophage [48, 49]. It is interesting to note that mircoRNA-containing exosomes were shown to be simultaneously released by MSCs under these conditions, acting to dampen TLR signalling when phagocytosed by recipient macrophages [49]. This suggests that given the appropriate co-signals, mtDAMPs are not always inflammatory and points to a therapeutic avenue for mircoRNA in post-injury hyperinflammation.

## Active release: neutrophil extracellular traps

Cf-mtDNA can be released by immune cells in the form of NETs. Blood from trauma patients and in other disease states such as sepsis, cancer, and SLE has been shown to contain neutrophil extracellular traps (NETs) [27, 50]. NETs are web-like structures of chromatin from DNA with interspersed histone and protein granules that are released by neutrophils to trap bacteria. In a trauma setting, NETosis triggered by non-infectious factors such DAMPs or platelet activation is thought to contribute to sterile inflammation that culminates in SIRS and multiple organ failure (MOF). Remarkably, cf-mtDNA has been shown to stimulate formation of NETs themselves [51]. The potential for self-perpetuating inflammation arises.

We are aware of at least two modes of NET formation, one demonstrated by Brinkman et al. termed ‘netosis’ which involves the death of the neutrophil upon NET formation and the other demonstrated by Yousefi et al. which involve viable neutrophils producing NETs [52, 53]. The remarkable contrast between the two modes is the source of chromatin in each. NETs from viable neutrophils contain only mtDNA whereas netosis produces a mixture of nuclear and mitochondrial DNA webs [52, 53]. Within neutrophils there are approximately 500 copies of mtDNA, hence abundant mitochondrial chromatin for NET formation [54].

## Methods of measuring cf-mtDNA

Reliable measurement of cf-mtDNA is essential to its study and utilisation as a clinical tool. Current research is aimed at understanding the natural history of mtDNA release after trauma with the present understanding that mtDNA release occurs rapidly after injury. The study of mtDNA relies on being able to accurately determine mtDNA release with ample sensitivity and specificity in a time-sensitive manner. The ideal method of measurement is quick, accurate, precise and based on easy-to-obtain samples. Most research is based on blood sampling with assays being run on plasma or serum.

Given the aforementioned forms that cf-mtDNA can take, different methods for sample processing could easily bias findings to reflect circulating DNA fragments rather than microparticle-derived mtDNA, for example. Centrifugation speeds used in cf-mtDNA studies are, therefore, key to the interpretation of results and future studies should be mindful of which cf-mtDNA components are being captured.

Quantitative polymerase chain reaction (qPCR) is the most commonly utilised cf-mtDNA assay. The test is typically run on plasma samples, separated from whole blood by means of centrifugation. This step removes cells, thus removing potential confounders in the form of intracellular mtDNA. Subsequently, plasma is centrifuged again at “high speed” to pellet cell debris. Importantly, the speed and time of this centrifugation step will determine which sources of cf-mtDNA are retained for analysis. Typically, microparticle and exosome isolation requires centrifugation forces or 20,000×*g* and 120,000×*g*, respectively, which is not standard practice in post-injury cf-mtDNA measurements. Therefore, it is likely that mtDNA-containing vesicles contribute to the cf-mtDNA load reported in such studies. However, since mitochondria themselves can vary greatly in size, the extent of extruded mitochondria-derived mDNA captured/lost during processing of trauma patient blood is entirely unknown.

After centrifugation, sample preparation can involve DNA extraction or be performed directly on plasma DNA extraction typically using a generic commercial kit for DNA purification from blood/tissue/cultured cells, where cells or any other membrane-encapsulated structures are lysed and all DNA in the sample is purified through binding to a positively charged resin such as silica. This process takes approximately 30 min. Alternatively, qPCR can also be done without extraction as outlined by Breitbach et al. [55], by simply diluting plasma. The value of this is not just cost saving, but also eliminates sample loss of fragmented DNA during extraction. Samples are analysed using a qPCR machine and DNA primers for one or more mitochondrial genes. Results are expressed as concentration in weight/volume or cycle threshold number. A result can typically be obtained within 3 h of sampling. qPCR can be run on any fluid sample to detect cf-mtDNA. Tissue sample detection is principally not as accurate for cf-mtDNA as sample processing will release intracellular mtDNA.

Spectrofluorometry is another method of detecting and quantifying cf-mtDNA. However, this method detects all DNA in the sample regardless of origin. Thus, it is not specific to mtDNA alone. Margraf et al. utilised this method by staining plasma sample DNA with PicoGreen. Cell-free DNA and NETs were visualised and quantified in weight/volume [56].

Flow cytometry is a fast, sensitive and specific test for quantifying mitochondria and microparticles. Specific markers for membrane-encapsulated mitochondria can be used in conjunction with cell-permeable mitochondrial stains such as Mitotracker to measure the cell-type origin [22, 57, 58]. For free mitochondria, outer membrane proteins such as TOM20 or TOM70 can be targeted for labelling [22].

Measurement of NETs has traditionally been achieved through staining of extruded DNA and/or citrullinated histones in conjunction with neutrophil-specific myeloperoxidase or neutrophil elastase and morphological identification using microscopy [52]. However, as a potential clinical biomarker, this is not quantitative, is laborious and is prone to observer bias. A number of studies have described methods for quantitating NETs with flow cytometry using a similar staining approach [59]. A major limitation of NET measurement in either case is the inability to specifically measure mtDNA within the structure (Table 1).

**Table 1:** Description of the capacity of four modalities of cf-mtDNA detection

Detection method	cf-mtDNA fragments	Mitochondria	Microparticles	NETs
qPCR	Specific	Non-specific	Non-specific	Non-specific
Flow cytometry	No	Specific	Specific	Specific
Spectrofluorometry	Non-specific	No	No	Non-specific
Microscopy	No	No	No	Specific

Specificity of each modality to detect the exact form of cf-mtDNA is described as specific or non-specific

## Mitochondrial DNA: pathways to inflammation

It appears that the inflammatory effects of mtDNA can be beneficial and harmful. Beneficial effects are seen with NET formation to fight invading microbes [53, 60]. In the trauma setting, the observation of high concentrations of cf-mtDNA and its association with multiple organ failure proposes a harmful scenario of mtDNA-induced inflammation [11]. It is understood that mtDNA can induce inflammation via a host of mechanisms. These can be simplified as immune activation via extracellular mtDNA interaction or via intracellular mtDNA interaction.

Intracellular mechanisms of mtDNA inflammation include inflammasome activation and stimulator of interferon gene pathway (STING) activation. These mechanisms have not been demonstrated directly in the trauma setting; however, plausible mechanisms exist based on available scientific research. Shimada et al. discovered mtDNA directly activates NLRP3 inflammasomes [41]. This interaction is first dependent on NLRP3 generation which occurs secondary to interleukin 1beta which has been shown to be elevated in polytrauma patients [41, 61]. This proposes a link between trauma NLRP3 generation, mtdNA activation of NLRP3 inflammasomes and subsequent inflammatory cytokine production.

Another intracellular mechanism of inflammation involving mtDNA is the STING pathway. The STING pathway is a mechanism of innate immunity towards cytosolic DNA that is beneficial in fighting infection [62, 63]. In trauma, however, cytosolic mtDNA can activate this pathway by binding to STING protein on endoplasmic reticulum. Cytosolic mtDNA activates cGAMP synthase resulting in downstream pro-inflammatory interferon secretion [64]. Interestingly, this also results in interleukin 1-beta production [63]. STING activation may then contribute to NLRP3 inflammasome generation, another mechanism of propagation of inflammation.

Cf-mtDNA has also been shown to interact with TLR9. This interaction can occur both extracellularly and intracellularly. The basis of TLR activation by mtDNA is the abundance of CpG motifs in mtDNA, a similarity it shares with bacterial DNA. TLR9 is expressed mainly not only within endolysosomes of cells but are also present on the surface of human PMNs [65, 66]. Thus, cf-mtDNA can interact with TLR9 extracellularly and intracellularly when released into the cytosol internally or internalised via microparticle engulfment. TLR9 interaction results in p38 MAPK activation and downstream pro-inflammatory cytokine release and NET formation [1, 16, 17, 51].

The innate immune response of NET formation has been described above and represents an example of extracellular mtDNA activation of inflammation. NETs formed in trauma have been shown to be predominantly made of mtDNA [50]. Itagaki et al. have shown that this phenomenon is TLR9 dependent [51].

## Clinical relevance and therapeutic targets

The potent pro-inflammatory nature of cf-mtDNA and its crucial role in the initiation phase of post-injury inflammation makes it an attractive therapeutic target. Efforts to decrease the initial tissue injury and shock-associated cell necrosis-driven cf-mtDNA release and sustained circulatory presence are seemingly obvious strategies. It is unknown how much cf-mtDNA is required to drive the essential and protective inflammatory response, which is required for the survival of the individual and recovery from injuries. A more attractive strategy is to modify secondary and mainly active mechanisms of cf-mtDNA release, especially in response to surgical interventions and other therapeutic events. Since many of these non-lifesaving secondary interventions can be planned and optimally timed, this approach presents a potential avenue towards precision medicine in tailoring definitive care to the needs of the injured individual.

Reducing cf-mtDNA is a promising avenue to prevent development of inflammatory complications of trauma. Traditional practice of debridement of necrotic tissue after trauma fits in with this thinking. Removal of necrotic tissue with its released cf-mtDNA reduces the total volume of cf-mtDNA that can enter circulation and interact with immune cells. However, this is not true for all surgical procedures and the nature of the surgery/procedure will likely determine the overall effect on cf-mtDNA release. As we know, procedures such as intramedullary nail fixation of long bone fractures are associated with higher cf-mtDNA concentrations and inflammatory cytokine release [67, 68]. This is seen as a second inflammatory hit following injury as the patient is subject to additional trauma in the form of intramedullary reaming. In other words, amputation of a mangled extremity is more likely to reduce cf-mtDNA while salvage and fixation is more likely to increase cf-mtDNA concentrations. Understanding the inflammatory repercussions of these interventions will help post-operative management.

There are also therapies aimed at reducing the harmful inflammatory effects of mtDNA. Several animal models have been utilised to study these therapies but clinical trials are lacking. Current strategies include therapies to reduce cf-mtDNA release, breakdown of released mtDNA and blocking of mtDNA binding receptors.

Yang et al. studied the effects of Endo III and Endonuclease III on mtDNA in a rat model of myocardial infarction. Endonuclease III is an enzyme that digests DNA while Endo III is a protein that facilitates the delivery of Endonuclease III into intracellular mitochondria. Their experiments found protective effects of the Endo III/Endonuclease III combination. A reduction of cf-mtDNA and reduction of myocardial infarct size were observed. This protective effect was found to be augmented by concomitant administration of Dnase I to digest circulating cf-mtDNA [31].

Dnase I is a pancreatic enzyme that is present in circulation under physiologic conditions and digests DNA [69]. Ming et al. observed a temporary rise in plasma Dnase I concentration on admission in polytrauma patients. The concentration normalised within 24 h [70]. Circulating cf-mtDNA is digested by Dnase I though only in vitro studies are available to prove this concept [38, 70]. Two studies have shown in vitro PMN activation by mtDNA with subsequent reduced activation by co-incubation with Dnase I [38, 70]. Intravenous administration is hypothesised to result in reduction of plasma cf-mtDNA while local administration of Dnase I to sites of injury at times of surgery (i.e fracture fixation) is hypothesised to reduce the volume of cf-mtDNA that reaches circulation.

With knowledge that ROS damage mitochondria and contribute to cf-mtDNA release, mitochondrial ROS-scavenging agents have been designed. Agents like MitoQ10 and Mito-VitE reduce ROS production [71]. Interestingly, Metformin has been shown to reduce ROS production in platelet concentrates. Metformin treatment of platelet concentrates reduced mtDNA release and protected platelet mitochondria from damage by ROS. The protective effect of Metformin is, however, lost if high concentrations of Metformin are used. High concentrations of Metformin stimulated platelet apoptosis with a resultant increase in mtDNA release [72].

Secondary active release of mtDNA from ROS exposure has been shown to be prevented by Cyclosporine A. Cyclosporine A prevents mitochondrial membrane pore formation and thus prevents release of mtDNA into the cytosol [73]. An area of study also exists for the use of monoclonal antibodies to manage sterile inflammation. Garcia-Martinez et al. and Zhang et al. developed TLR9 antagonists to block the binding of cf-mtDNA to TLR9. They observed a protective effect of TLR9 against cf-mtDNA-induced inflammation in non-alcoholic steatohepatitis and p38 MAPK activation, respectively [16, 44]. It is unclear how effective each agent will be in managing inflammation after trauma. Clinical trials are required. It is plausible that a combination of multiple agents will have better suppression of cf-mtDNA and overall reduction of inflammatory complications.

## Conclusion

Research on cf-mtDNA in trauma is growing but still insufficient to change clinical management. Mitochondria are organelles critical to survival and disruption of their function and integrity leads to inflammation and tissue injury. Cf-mtDNA originates from the abundant mitochondria in cells via complex mechanisms of passive and active release. Multiple harmful effects are implicated from high concentrations of cf-mtDNA and efforts are underway to understand its natural history and relevance to severity of injury. Simultaneous efforts to develop therapeutic agents that neutralise cf-mtDNA are ongoing. Goals of mtDNA research in trauma are to determine validity of cf-mtDNA as a measure of injury severity, predictor of SIRS/MOF and guide to optimal timing of surgical management. The development of therapeutic agents to neutralise the inflammatory effects of cf-mtDNA promises to dramatically augment trauma management.

## References

[1] Gu X, Wu G, Yao Y, Zeng J, Shi D, Lv T (2015). Intratracheal administration of mitochondrial DNA directly provokes lung inflammation through the TLR9-p38 MAPK pathway. Free Radic Biol Med.

[2] Xie L, Liu S, Cheng J, Wang L, Liu J, Gong J (2017). Exogenous administration of mitochondrial DNA promotes ischemia reperfusion injury via TLR9-p38 MAPK pathway. Regul Toxicol Pharmacol RTP.

[3] Zhang JZ, Wang J, Qu WC, Wang XW, Liu Z, Ren JX (2017). Plasma mitochondrial DNA levels were independently associated with lung injury in elderly hip fracture patients. Injury.

[4] Hu Q, Ren J, Wu J, Li G, Wu X, Liu S (2017). Elevated levels of plasma mitochondrial DNA are associated with clinical outcome in intra-abdominal infections caused by severe trauma. Surg Infect.

[5] Krychtiuk K, Wurm R, Ruhittel S, Lenz M, Huber K, Wojta J, et al. Mitochondrial DNA predicts mortality in acute but not in chronic heart failure. Intensive Care Medicine Experimental Conference: 30th Annual Congress of the European Society of Intensive Care Medicine, ESICM. 2017;5(2 Supplement 1).

[6] Sheen D, Yoo J, Kim SA, Lim MK (2017). Association of mitochondrial DNA copy number with disease activity in rheumatoid arthritis. Int J Rheum Dis.

[7] Simula L, Nazio F, Campello S (2017). The mitochondrial dynamics in cancer and immune-surveillance. Semin Cancer Biol.

[8] Cicchillitti L, Corrado G, de Angeli M, Mancini E, Baiocco E, Patrizi L (2017). Circulating cell-free DNA content as blood based biomarker in endometrial cancer. Oncotarget.

[9] Li L, Hann HW, Wan S, Hann RS, Wang C, Lai Y (2016). Cell-free circulating mitochondrial DNA content and risk of hepatocellular carcinoma in patients with chronic HBV infection. Sci Rep.

[10] Tuboly E, McLlroy D, Briggs G, Lott N, Balogh ZJ (2017). Clinical implications and pathological associations of circulating mitochondrial DNA. Front Biosci (Landmark edn).

[11] Simmons JD, Lee YL, Mulekar S, Kuck JL, Brevard SB, Gonzalez RP (2013). Elevated levels of plasma mitochondrial DNA DAMPs are linked to clinical outcome in severely injured human subjects. Ann Surg.

[12] Skulachev VP (1999). Mitochondrial physiology and pathology; concepts of programmed death of organelles, cells and organisms. Mol Asp Med.

[13] West AP, Shadel GS (2017). Mitochondrial DNA in innate immune responses and inflammatory pathology. Nat Rev Immunol.

[14] Osellame LD, Blacker TS, Duchen MR (2012). Cellular and molecular mechanisms of mitochondrial function. Best Pract Res Clin Endocrinol Metab.

[15] Wilkins HM, Weidling IW, Ji Y, Swerdlow RH (2017). Mitochondria-derived damage-associated molecular patterns in neurodegeneration. Front Immunol.

[16] Zhang Q, Raoof M, Chen Y, Sumi Y, Sursal T, Junger W (2010). Circulating mitochondrial DAMPs cause inflammatory responses to injury. Nature.

[17] Zhang Q, Itagaki K, Hauser CJ (2010). Mitochondrial DNA is released by shock and activates neutrophils via p38 map kinase. Shock (Augusta Ga).

[18] Manson J, Thiemermann C, Brohi K (2012). Trauma alarmins as activators of damage-induced inflammation. Br J Surg.

[19] Alam TI, Kanki T, Muta T, Ukaji K, Abe Y, Nakayama H (2003). Human mitochondrial DNA is packaged with TFAM. Nucleic Acids Res.

[20] Mengel-From J, Thinggaard M, Dalgard C, Kyvik KO, Christensen K, Christiansen L (2014). Mitochondrial DNA copy number in peripheral blood cells declines with age and is associated with general health among elderly. Hum Genet.

[21] Ye F, Samuels DC, Clark T, Guo Y (2014). High-throughput sequencing in mitochondrial DNA research. Mitochondrion.

[22] Boudreau LH, Duchez AC, Cloutier N, Soulet D, Martin N, Bollinger J (2014). Platelets release mitochondria serving as substrate for bactericidal group IIA-secreted phospholipase A2 to promote inflammation. Blood.

[23] Bereiter-Hahn J, Voth M (1994). Dynamics of mitochondria in living cells: shape changes, dislocations, fusion, and fission of mitochondria. Microsc Res Tech.

[24] Elmore S, Apoptosis (2007). A review of programmed cell death. Toxicol Pathol.

[25] Lemasters JJ (2014). Variants of mitochondrial autophagy: types 1 and 2 mitophagy and micromitophagy (Type 3). Redox Biol.

[26] Lam NY, Rainer TH, Chiu RW, Joynt GM, Lo YM (2004). Plasma mitochondrial DNA concentrations after trauma. Clin Chem.

[27] McIlroy DJ, Bigland M, White AE, Hardy BM, Lott N, Smith DW (2015). Cell necrosis-independent sustained mitochondrial and nuclear DNA release following trauma surgery. J Trauma Acute Care Surg.

[28] Yamanouchi S, Kudo D, Yamada M, Miyagawa N, Furukawa H, Kushimoto S (2013). Plasma mitochondrial DNA levels in patients with trauma and severe sepsis: time course and the association with clinical status. J Crit Care.

[29] Rock KL, Kono H (2008). The inflammatory response to cell death. Ann Rev Pathol.

[30] Gu X, Yao Y, Wu G, Lv T, Luo L, Song Y (2013). The plasma mitochondrial DNA Is an Independent predictor for post-traumatic systemic inflammatory response syndrome. PloS One.

[31] Yang XM, Cui L, White J, Kuck J, Ruchko MV, Wilson GL (2015). Mitochondrially targeted Endonuclease III has a powerful anti-infarct effect in an in vivo rat model of myocardial ischemia/reperfusion. Basic Res Cardiol.

[32] Qin CY, Zhang HW, Gu J, Xu F, Liang HM, Fan KJ (2017). Mitochondrial DNA induced inflammatory damage contributes to myocardial ischemia reperfusion injury in rats: cardioprotective role of epigallocatechin. Mol Med Rep.

[33] Martinez-Quinones PA, McCarthy CG, Mentzer CJ, Wenceslau CF, Holsten SB, Webb RC (2017). Peritoneal cavity lavage reduces the presence of mitochondrial damage associated molecular patterns in open abdomen patients. J Trauma Acute Care Surg.

[34] Hauser CJ, Sursal T, Rodriguez EK, Appleton PT, Zhang Q, Itagaki K (2010). Mitochondrial damage associated molecular patterns from femoral reamings activate neutrophils through formyl peptide receptors and P44/42 MAP kinase. J Orthop Trauma.

[35] Sandler N, Kaczmarek E, Itagaki K, Zheng Y, Otterbein L, Khabbaz K (2018). Mitochondrial DAMPs are released during cardiopulmonary bypass surgery and are associated with postoperative atrial fibrillation. Heart Lung Circ.

[36] McQueen A, Meilhoc E, Bailey JE (1987). Flow effects on the viability and lysis of suspended mammalian cells. Biotechnol Lett.

[37] Casa LDC, Deaton DH, Ku DN (2015). Role of high shear rate in thrombosis. J Vasc Surg.

[38] Puyo CA, Peruzzi D, Earhart A, Roller E, Karanikolas M, Kollef MH (2017). Endotracheal tube-induced sore throat pain and inflammation is coupled to the release of mitochondrial DNA. Mol Pain.

[39] Larsen NB, Rasmussen M, Rasmussen LJ (2005). Nuclear and mitochondrial DNA repair: similar pathways?. Mitochondrion.

[40] Caielli S, Athale S, Domic B, Murat E, Chandra M, Banchereau R (2016). Oxidized mitochondrial nucleoids released by neutrophils drive type I interferon production in human lupus. J Exp Med.

[41] Shimada K, Crother TR, Karlin J, Dagvadorj J, Chiba N, Chen S (2012). Oxidized mitochondrial DNA activates the NLRP3 inflammasome during apoptosis. Immunity.

[42] Lamkanfi M (2011). Emerging inflammasome effector mechanisms. Nat Rev Immunol.

[43] Gyorgy B, Szabo TG, Pasztoi M, Pal Z, Misjak P, Aradi B (2011). Membrane vesicles, current state-of-the-art: emerging role of extracellular vesicles. Cell Mol Life Sci CMLS.

[44] Garcia-Martinez I, Santoro N, Chen Y, Hoque R, Ouyang X, Caprio S (2016). Hepatocyte mitochondrial DNA drives nonalcoholic steatohepatitis by activation of TLR9. J Clin Invest.

[45] Mobarrez FFE, Gunnarsson I, Pisetsky D, Svenungsson E. The expression of mitochondrial molecules in microparticle immune complexes in the blood of patients with systemic lupus erythematosus. Arthritis Rheumatol. 2017;69(suppl 10). http://acrabstracts.org/abstract/the-expression-of-mitochondrial-molecules-in-microparticle-immune-complexes-in-the-blood-of-patients-with-systemic-lupus-erythematosus. Accessed 5 Jan 2018

[46] Marcoux G, Duchez AC, Rousseau M, Levesque T, Boudreau LH, Thibault L (2017). Microparticle and mitochondrial release during extended storage of different types of platelet concentrates. Platelets.

[47] Duchez A-C, Boudreau LH, Naika GS, Bollinger J, Belleannée C, Cloutier N (2015). Platelet microparticles are internalized in neutrophils via the concerted activity of 12-lipoxygenase and secreted phospholipase A < sub > 2-IIA. Proc Natl Acad Sci.

[48] Maeda A, Fadeel B (2014). Mitochondria released by cells undergoing TNF-alpha-induced necroptosis act as danger signals. Cell Death Dis.

[49] Phinney DG, Di Giuseppe M, Njah J, Sala E, Shiva S, St Croix CM (2015). Mesenchymal stem cells use extracellular vesicles to outsource mitophagy and shuttle microRNAs. Nat Commun.

[50] McIlroy DJ, Jarnicki AG, Au GG, Lott N, Smith DW, Hansbro PM (2014). Mitochondrial DNA neutrophil extracellular traps are formed after trauma and subsequent surgery. J Crit Care.

[51] Itagaki K, Kaczmarek E, Lee YT, Tang IT, Isal B, Adibnia Y (2015). Mitochondrial DNA released by trauma induces neutrophil extracellular traps. PloS One.

[52] Brinkmann V, Reichard U, Goosmann C, Fauler B, Uhlemann Y, Weiss DS (2004). Neutrophil extracellular traps kill bacteria. Science.

[53] Yousefi S, Mihalache C, Kozlowski E, Schmid I, Simon HU (2009). Viable neutrophils release mitochondrial DNA to form neutrophil extracellular traps. Cell Death Diff.

[54] Satoh M, Kuroiwa T (1991). Organization of multiple nucleoids and DNA molecules in mitochondria of a human cell. Exp Cell Res.

[55] Breitbach S, Tug S, Helmig S, Zahn D, Kubiak T, Michal M (2014). Direct quantification of cell-free, circulating DNA from unpurified plasma. PloS One.

[56] Margraf S, Logters T, Reipen J, Altrichter J, Scholz M, Windolf J (2008). Neutrophil-derived circulating free DNA (cf-DNA/NETs): a potential prognostic marker for posttraumatic development of inflammatory second hit and sepsis. Shock (Augusta Ga).

[57] De Paoli SH, Tegegn TZ, Elhelu OK, Strader MB, Patel M, Diduch LL (2018). Dissecting the biochemical architecture and morphological release pathways of the human platelet extracellular vesiculome. Cell Mol Life Sci CMLS.

[58] Chou SH-Y, Lan J, Esposito E, Ning M, Balaj L, Ji X (2017). Extracellular mitochondria in cerebrospinal fluid and neurological recovery after subarachnoid hemorrhage. Stroke.

[59] Masuda S, Nakazawa D, Shida H, Miyoshi A, Kusunoki Y, Tomaru U (2016). NETosis markers: quest for specific, objective, and quantitative markers. Clin Chim Acta.

[60] Yousefi S, Gold JA, Andina N, Lee JJ, Kelly AM, Kozlowski E (2008). Catapult-like release of mitochondrial DNA by eosinophils contributes to antibacterial defense. Nat Med.

[61] Shahbazian LM, Jeevanandam M, Petersen SR (1999). Release of proinflammatory cytokines by mitogen-stimulated peripheral blood mononuclear cells from critically ill multiple-trauma victims. Metab Clin Exp.

[62] Barber GN (2014). STING-dependent cytosolic DNA sensing pathways. Trends Immunol.

[63] Sun L, Wu J, Du F, Chen X, Chen ZJ (2013). Cyclic GMP-AMP synthase is a cytosolic DNA sensor that activates the type-I interferon pathway. Science (New York, NY).

[64] West AP, Khoury-Hanold W, Staron M, Tal MC, Pineda CM, Lang SM (2015). Mitochondrial DNA stress primes the antiviral innate immune response. Nature.

[65] Barton GM, Kagan JC, Medzhitov R (2006). Intracellular localization of Toll-like receptor 9 prevents recognition of self DNA but facilitates access to viral DNA. Nat Immunol.

[66] Eaton-Bassiri A, Dillon SB, Cunningham M, Rycyzyn MA, Mills J, Sarisky RT (2004). Toll-like receptor 9 can be expressed at the cell surface of distinct populations of tonsils and human peripheral blood mononuclear cells. Infect Immun.

[67] Gan L, Zhong J, Zhang R, Sun T, Li Q, Chen X (2015). The immediate intramedullary nailing surgery increased the mitochondrial DNA release that aggravated systemic inflammatory response and lung injury induced by elderly hip fracture. Mediat Inflamm.

[68] Pape HC, Grimme K, Van Griensven M, Sott AH, Giannoudis P, Morley J (2003). Impact of intramedullary instrumentation versus damage control for femoral fractures on immunoinflammatory parameters: prospective randomized analysis by the EPOFF study group. J Trauma.

[69] Love JD, Hewitt RR (1979). The relationship between human serum and human pancreatic DNase I. J Biol Chem.

[70] Meng W, Paunel-Gorgulu A, Flohe S, Witte I, Schadel-Hopfner M, Windolf J (2012). Deoxyribonuclease is a potential counter regulator of aberrant neutrophil extracellular traps formation after major trauma. Mediat Inflamm.

[71] Li X, Fang P, Mai J, Choi ET, Wang H, Yang X (2013). Targeting mitochondrial reactive oxygen species as novel therapy for inflammatory diseases and cancers. J Hematol Oncol.

[72] Xin G, Wei Z, Ji C, Zheng H, Gu J, Ma L (2016). Metformin uniquely prevents thrombosis by inhibiting platelet activation and mtDNA release. Sci Rep.

[73] Halestrap AP, Davidson AM (1990). Inhibition of Ca2(+)-induced large-amplitude swelling of liver and heart mitochondria by cyclosporin is probably caused by the inhibitor binding to mitochondrial-matrix peptidyl-prolyl cis-trans isomerase and preventing it interacting with the adenine nucleotide translocase. Biochem J.

